# Factors associated with long‐term HIV pre‐exposure prophylaxis engagement and adherence among transgender women in Brazil, Mexico and Peru: results from the ImPrEP study

**DOI:** 10.1002/jia2.25974

**Published:** 2022-10-12

**Authors:** Kelika A. Konda, Thiago S. Torres, Gabriela Mariño, Alessandra Ramos, Ronaldo I. Moreira, Iuri C. Leite, Marcelo Cunha, Emilia M. Jalil, Brenda Hoagland, Juan V. Guanira, Marcos Benedetti, Cristina Pimenta, Heleen Vermandere, Sergio Bautista‐Arredondo, Hamid Vega‐Ramirez, Valdilea G. Veloso, Carlos F. Caceres, Beatriz Grinsztejn, for the ImPrEP Study Group

**Affiliations:** ^1^ Universidad Peruana Cayetano Heredia, Centro de Investigaciones Interdisciplinaria en Salud, Sexualidad, y SIDA Lima Peru; ^2^ Instituto Nacional de Infectologia Evandro Chagas, Fundação Oswaldo Cruz (INI‐Fiocruz) Rio de Janeiro Brazil; ^3^ Escola Nacional de Saúde Pública, Fundação Oswaldo Cruz (ENSP‐Fiocruz) Rio de Janeiro Brazil; ^4^ Ministério da Saúde Brasília Brazil; ^5^ Instituto Nacional de Salud Pública (INSP) Cuernavaca Mexico; ^6^ Instituto Nacional de Psiquiatria Ramon de la Fuente Muñiz Mexico City Mexico

**Keywords:** pre‐exposure prophylaxis, transgender persons, HIV, Latin America, medication adherence, public health

## Abstract

**Introduction:**

The HIV epidemic continues to disproportionately impact Latin‐American transgender women (TGW). We assessed factors associated with long‐term pre‐exposure prophylaxis (PrEP) engagement and adherence among TGW enrolled in the Implementation of PrEP (ImPrEP) study, the largest PrEP demonstration study in Latin America.

**Methods:**

HIV‐negative TGW aged ≥18 years reporting 1+eligibility criteria in the 6 months prior to enrolment (e.g. sex partner known to be living with HIV, condomless anal sex [CAS], transactional sex or having a sexually transmitted infection [STI]) who could safely take PrEP were enrolled. Follow‐up visits were conducted at 4 weeks and then quarterly. We conducted logistic regression to identify factors associated with long‐term PrEP engagement (3+ follow‐up visits in 52 weeks) and complete self‐reported adherence (no missed pills in the past 30 days) during follow‐up. For both outcomes, we constructed multivariable models controlling for country, socio‐demographics, sexual behaviour, substance use, STIs and self‐reported adherence at 4 weeks (long‐term engagement outcome only).

**Results:**

From March 2018 to June 2021, ImPrEP screened 519 TGW, enrolled 494 (Brazil: 190, Mexico: 66 and Peru: 238) and followed them for 52 weeks. At baseline, 27.5% of TGW were aged 18–24 years, 67.8% were mixed‐race and 31.6% had >secondary education. Most, 89.9% reported CAS, 61.9% had >10 sex partners and 71.9% reported transactional sex. HIV incidence was 1.82 cases per 100 person‐years (95% confidence interval [CI]: 0.76–4.38). Almost half of TGW (48.6%) had long‐term PrEP engagement, which was positively associated with reporting complete adherence at week 4 (aOR:2.94 [95%CI:1.88–4.63]) and was inversely associated with reporting CAS with unknown‐HIV partner (aOR:0.52 [95%CI:0.34–0.81]), migration (aOR:0.54 [95%CI:0.34–0.84]), and being from Mexico (aOR:0.28 [95%CI:0.14–0.53]). Self‐reported adherence was associated with TGW aged >34 (aOR:1.61 [95%CI:1.10–2.34]) compared to those aged 25–34 and those with >secondary education (aOR:1.55 [95%CI:1.10–2.19]) and was lower among TGW from Peru (aOR:0.29 [95%CI:0.21–0.41]) or reporting PrEP‐related adverse effects (aOR:0.63 [95%CI:0.42–0.92]).

**Conclusions:**

Although TGW were willing to enrol in ImPrEP, long‐term PrEP engagement and complete self‐reported adherence were limited, and HIV incidence remained relatively high. A successful HIV prevention agenda should include trans‐specific interventions supporting oral PrEP and exploring long‐acting PrEP strategies for TGW.

## 1 INTRODUCTION

HIV infection disproportionately impacts transgender women (TGW) worldwide, with HIV prevalence being 50 times greater than adults of reproductive age in low‐ and middle‐income countries (LMICs), such as those in Latin America [1, 2, 3]. The HIV prevalence among TGW in Latin America was estimated at 25.9% [4], 32–49% in Brazil [5], 20–64% in Mexico [6] and 30% in Peru [7]. This increased vulnerability is caused by substantial social marginalization and isolation experienced by TGW, leading to poverty, lower education and exclusion from the formal labour market [8], leading to high rates of sex work [6, 9, 10, 11]. In Brazil, Mexico and Peru, TGW also experience substantial violence [11, 12, 13, 14], internalized stigma and fear of discrimination [15, 16] and increased burdens of mental health and substance abuse [17]. These vulnerabilities can also influence their health‐seeking behaviour and engagement in HIV prevention services. Moreover, these services often do not have the resources to truly address the needs of this population [18].

Daily oral pre‐exposure prophylaxis (PrEP) with tenofovir disoproxil fumarate 300 mg (TDF) combined with emtricitabine 200 mg (FTC) has been demonstrated to prevent HIV infection [19]. Still, it is highly dependent on pill adherence and engagement in prevention services [20, 21]. A sub‐analysis of TGW included in the iPrEx study yielded no difference in HIV acquisition between study arms (PrEP vs. placebo); however, PrEP was efficacious in preventing HIV among TGW who were adherent to daily oral PrEP as measured by drug levels [22]. Questions remain on the interactions between feminizing hormone therapy (FHT) and PrEP among TGW, with studies showing decreased levels of TDF/FTC among FHT users [23, 24, 25], or lack of interaction [26]. The vulnerability of TGW to HIV makes their use of PrEP of vital importance [27, 28, 29]. However, few TGW have been engaged in HIV prevention services [5, 30] or PrEP studies [31], hindering the possibility of meaningful analysis [32], despite high willingness to use PrEP [33, 34, 35]. In addition, PrEP studies have shown low PrEP continuation among TGW [36]. Research has highlighted the need for PrEP programmes to specifically address the needs of trans populations, including TGW [31, 37, 38, 39]. However, efforts towards this end have been limited [3].

Although daily oral PrEP was recommended in 2014 by the World Health Organization, PrEP availability has been limited in Latin America [29, 40]. PrEP has been available within Brazil's Public Health System (SUS) since 2017, Mexico since 2021 [41], but remains accessible only via purchase or through limited demonstration studies in Peru. The Implementation of PrEP (ImPrEP) study is the largest PrEP demonstration study in Latin America and aims to evaluate the feasibility of PrEP implementation among gay, bisexual and other cisgender men who have sex with men (MSM) and TGW in the context of the Public Health Systems of Brazil, Mexico and Peru. This analysis aims to assess the factors associated with long‐term PrEP engagement and self‐reported adherence among TGW enrolled in the ImPrEP study.

## 2 METHODS

### 2.1 Study design and participants

ImPrEP was a prospective, single arm, open‐label, multicentre study that assessed same‐day oral PrEP implementation in Brazil (14 sites in 12 cities), Mexico (4 sites in 3 cities) and Peru (10 sites in 6 cities). Inclusion criteria were HIV‐negative MSM and TGW, aged ≥18 years and at least one of the following in the prior 6 months: condomless anal sex (CAS), anal sex with partner(s) known to be living with HIV, sexual transmitted infections (STIs) signs/symptoms or diagnosis, or transactional sex. Participants were enrolled from March 2018 to December 2020. This analysis only includes participants self‐identified as women, *travestis* [12, 33, 42] or TGW who had time to complete 52 weeks of follow‐up by 30th June 2021 (data extraction).

Institutional review board (IRB) in each country approved the study: in Brazil, INI Evandro Chagas‐FIOCRUZ IRB (#CAAE:79259517.5.1001.5262) and local IRB at each Brazilian site; in Mexico, National Institute of Public Health IRB (#CI‐1515); and in Peru, Universidad Peruana Cayetano Heredia IRB (#100740). All study participants provided written informed consent before initiating any study procedure. The study was registered at the Brazilian Registry of Clinical Trials (ReBEC:20‐Aug‐2018, ID RBR‐4×3cnp, UTN code: U1111‐1217‐6021).

### 2.2 Study procedures

Participants were recruited through social media advertisements, peer/healthcare provider referrals and through MSM/TGW peer‐educators at each site. We also offered enrolment to individuals seeking PrEP or HIV/STI testing. Potentially eligible individuals were screened using laboratory, clinical and risk criteria and enrolled to receive same‐day oral PrEP [43]. HIV viral load and serum creatinine clearance (CrCl) were evaluated at enrolment. Participants were contacted to discontinue PrEP and return to the site in case of acute HIV infection (detectable HIV viral load) or CrCl<60 ml/minute [44]. Follow‐up visits were scheduled at week 4 and quarterly thereafter, for a total of five planned follow‐up visits in 52 weeks. Given restrictions due to the COVID‐19 pandemic during 2020 and 2021 [45, 46, 47], the total number of visits and the visit intervals were impacted. At each visit, participants received TDF/FTC refills according to the next scheduled visit interval. Individuals who returned more than 24 weeks after any visit were required to re‐enrol in the study.

Data on demographics, prior post‐exposure prophylaxis (PEP) use (past 12 months), indication for PEP and the main reason for attending the service were assessed at enrolment. Participants also reported information on sexual behaviour and substance use at enrolment and quarterly visits. Self‐reported adherence and symptoms related to PrEP use were assessed at follow‐up visits. HIV rapid tests were performed every visit; HIV confirmatory testing was conducted as needed.

### 2.3 Study definitions

Age was described as median and interquartile range (IQR) and in categorical ranges of 18–24, 25–34 and >34 years. We categorized self‐reported race/skin colour as White, Black, Indigenous, Asian and Mixed‐race (*Pardo* or *Mestizo*); however, as these categories are distinct by country, they were dichotomized into white versus any other race. We used the following education categories: primary or less (complete or incomplete), secondary (complete or incomplete) and more than secondary. Individuals born in a state or country different from the implementation site were considered as migrants. Main reason to attend the service was stratified as seeking PrEP and other (seeking an HIV test, other health service or PEP).

Sexual behaviour was assessed with the questions: number of cisgender men or/and TGW sexual partners (described with median and IQR, categorized into <5, 5–10 and >10 for analyses), any CAS (yes/no), receptive CAS (yes/no), CAS with partner(s) known to be living with HIV (yes, no or I don't know) and transactional sex (sex in exchange for money, drugs, gifts or favours; yes/no). Binge drinking was assessed with the question: “Did you have five or more drinks within a two‐hour period?” (yes/no) [48]. Stimulant use was considered use of any of the following: club drugs (e.g. ecstasy, LSD and GHB), cocaine (powder, crack or base). PrEP‐related gastrointestinal symptoms were defined as any of the following: diarrhoea, flatulence, nausea, vomit, abdominal pain or other. At enrolment, questions on sexual behaviour referred to the previous 6 months, while number of sex partners in Brazil and Mexico and substance use referred to the previous 3 months. At quarterly visits, all questions referred to the previous 3 months. At the 4‐week visit, any PrEP‐related symptom(s) referred to the previous 30 days; at other visits, this information dated back to the period since the last visit.

### 2.4 Outcomes

We evaluated two main outcomes: long‐term PrEP engagement and complete self‐reported adherence. Long‐term PrEP engagement was defined as attendance at the 4‐week visit and two or more quarterly visits within a 52‐week period. As most participants attending these three visits would have received 210 PrEP pills (30 pills at enrolment and 90 pills at each follow‐up visit), this would be enough for achieving highly protective levels of tenofovir diphosphate (4 pills per week for 52 weeks) [20]. Participants’ self‐reported adherence was assessed at every follow‐up visit with the question: “In the previous 30 days, approximately how many pills did you NOT take?” Those who answered zero were considered as having complete self‐reported adherence, as a previous analysis estimated “zero” as the self‐reported PrEP adherence cut‐off equivalent to highly protective levels of tenofovir diphosphate [49, 50]. Individuals who re‐enrolled in the study completed the initial study assessment, which did not include an adherence question. Re‐enrolled individuals were classified as non‐adherent as the quantity of pills received in their prior visit (30 or 90) would have been insufficient to cover the period that they were absent from the study.

### 2.5 Statistical analysis

We described TGW's characteristics at enrolment, long‐term PrEP engagement and self‐reported adherence overall and according to country. We censored participants at study withdrawal or on 30th June 2021. HIV incidence was calculated based on the number of new HIV cases detected during the follow‐up overall and stratified by country and age.

We used logistic regression to identify initial enrolment factors associated with long‐term PrEP engagement. Potential predictors included baseline socio‐demographic and behavioural characteristics, such as country, age group, race, education, main reason to attend the service, migration, number of sex partners, any CAS, receptive CAS, CAS with partner known to be living with HIV, transactional sex, binge drinking, stimulant use and self‐reported adherence at week 4. Individuals who did not return to follow‐up visits were considered non‐adherent. We evaluated PrEP‐related gastrointestinal symptoms in bivariate analysis, but not in the multivariable model as this variable is only available for individuals returning to a week 4 visit, which would modify the analytic sample. In the initial model, the effect of each variable was controlled by country and all statistically significant variables at a *p*‐value ≤0.1 were included in the final adjusted model.

To account for correlated measures within participants, we used logistic generalized estimating equation models to identify factors associated with complete self‐reported adherence at each post‐enrolment visit completed by the study participants over the 52 weeks. We used the same potential predictors considered in the long‐term PrEP engagement analysis allowing behavioural characteristics and symptoms related to PrEP to be included as time‐varying variables. In the initial models, the effect of each variable was controlled by country and study visit. All variables statistically significant at *p*‐value ≤0.1 were included in the final adjusted model. All analyses were conducted in R version 4.1.1 [51].

## 3 RESULTS

**Figure 1:**
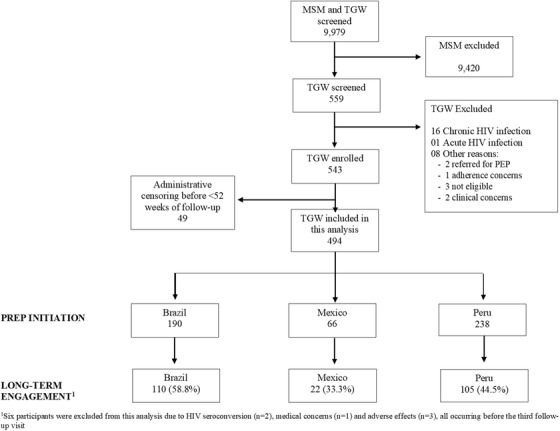
Study flow chart. Abbreviations: MSM, men who have sex with men; PrEP, pre‐exposure prophylaxis: TGW, transgender women.

A total of 9979 individuals were screened, 559 (5.6%) TGW. Of these, 543 were enrolled and 494 were followed for at least 52 weeks and included in this analysis (Brazil: 190, Mexico: 66 and Peru: 238) (Figure 1). Reasons for ineligibility included HIV infection at screening/enrolment (one acute and 16 chronic HIV infections), referral for PEP, adherence concerns (clinician thought the person would not be adherent to PrEP) and clinical concerns (other clinical condition, such as untreated tuberculosis or diabetes) (Figure 1). During follow‐up, 32 individuals were re‐enrolled, their additional visits were included in our analysis.

Among the 494 TGW included in this analysis, median age was 29 years (IQR: 24, 36); 27.5% aged 18–24 years. Most were mixed race (67.8%), had secondary education (58.7%), had not migrated (70.2%), attended the service seeking PrEP (65.4%) and most (71.9%) reported transactional sex. Median number of sex partners was 25 (IQR: 5, 100), and 61.9% reported >10 partners. The majority reported CAS (89.9%) and CAS with partner with unknown HIV status (64.8%), while 4.0% reported CAS with partner known to be living with HIV. Binge drinking and stimulant use were reported by 67.8% and 20.2%, respectively (Table 1).

**Table 1:** Characteristics at enrolment, long‐term PrEP engagement and complete self‐reported adherence at week 4 among TGW according to country

	Total	Brazil	Mexico	Peru	
	*N* = 494 (%)	*N* = 190 (%)	*N* = 66 (%)	*N* = 238 (%)	*p* Value^h^
Age (Years)					0.013
Median (IQR)	29 (24, 36)	28 (23, 34)	28 (24, 34)	31 (25, 38)	
18–24	136 (27.5)	62 (32.6)	19 (28.8)	55 (23.1)	
25–34	208 (42.1)	84 (44.2)	31 (47.0)	93 (39.1)	
>34	150 (30.4)	44 (23.2)	16 (24.2)	90 (37.8)	
Race or skin colour					<0.001
White	106 (21.5)	70 (36.8)	6 (9.1)	30 (12.6)	
Black	42 (8.5)	21 (11.1)	2 (3.0)	19 (8.0)	
Mixed‐race (*Pardo* or *Mestizo*)	335 (67.8)	95 (50.0)	58 (87.9)	182 (76.5)	
Asian	2 (0.4)	2 (1.1)	0 (0.0)	0 (0.0)	
Indigenous	9 (1.8)	2 (1.1)	0 (0.0)	7 (2.9)	
Education					
Primary (complete or incomplete)	48 (9.7)	20 (10.5)	7 (10.6)	21 (8.8)	<0.001
Secondary (complete or incomplete)	290 (58.7)	106 (55.8)	22 (33.3)	162 (68.1)	
More than secondary	156 (31.6)	64 (33.7)	37 (56.1)	55 (23.1)	
Gender identity					<0.001
Transgender woman	377 (76.3)	130 (68.4)	64 (97.0)	183 (76.9)	
*Travesti*	66 (13.4)	34 (17.9)	1 (1.5)	31 (13.0)	
Woman	51 (10.3)	26 (13.7)	1 (1.5)	24 (10.1)	
Migration					0.010
Yes	141 (29.8)	51 (29.1)	10 (15.4)	80 (34.3)	
No	332 (70.2)	124 (70.9)	55 (84.6)	153 (65.7)	
Main reason to attend the service					<0.001
Seeking PrEP	323 (65.4)	177 (93.2)	61 (92.4)	85 (35.7)	
Other	171 (34.6)	13 (6.8)	5 (7.6)	153 (64.3)	
PEP use^a^					<0.001
Yes	64 (13.0)	55 (28.9)	8 (12.1)	1 (0.4)	
No	430 (87.0)	135 (71.1)	58 (87.9)	237 (99.6)	
Number of cisgender men or/and TGW sex partners^b^					0.026
Median (IQR)	25 (5, 100)	33 (5, 158)	20 (9, 60)	20 (5, 60)	
<5	104 (21.1)	39 (20.5)	7 (10.6)	58 (24.4)	
5–10	84 (17.0)	26 (13.7)	18 (27.3)	40 (16.8)	
>10	306 (61.9)	125 (65.8)	41 (62.1)	140 (58.8)	
CAS^c^					
Yes	444 (89.9)	168 (88.4)	53 (80.3)	223 (93.7)	0.006
No	50 (10.1)	22 (11.6)	13 (19.7)	15 (6.3)	
Receptive CAS^c^					
Yes	424 (85.8)	160 (84.2)	50 (75.8)	214 (89.9)	0.011
No	70 (14.2)	30 (15.8)	16 (24.2)	24 (10.1)	
CAS with partner known to be living with HIV^c^					0.400
Yes	20 (4.0)	11 (5.8)	3 (4.5)	6 (2.5)	
No	154 (31.2)	54 (28.4)	21 (31.8)	79 (33.2)	
I don't know	320 (64.8)	125 (65.8)	42 (63.6)	153 (64.3)	
Transactional sex^c^					0.200
Yes	355 (71.9)	133 (70.0)	54 (81.8)	168 (70.6)	
No	139 (28.1)	57 (30.0)	12 (18.2)	70 (29.4)	
Binge drinking^d^					<0.001
Yes	333 (67.7)	114 (60.6)	34 (51.5)	185 (77.7)	
No	159 (32.3)	74 (39.4)	32 (48.5)	53 (22.3)	
Stimulant use^d^, ^e^					0.002
Yes	100 (20.2)	48 (25.3)	19 (28.8)	33 (13.9)	
No	394 (79.8)	142 (74.7)	47 (71.2)	205 (86.1)	
Long‐term PrEP engagement^f^					<0.001
Yes	237 (48.6)	110 (58.8)	22 (33.3)	105 (44.5)	
No	251 (51.4)	77 (41.2)	44 (66.7)	131 (55.5)	
Early continuation (attending 4‐week visit within the initial 60 days of follow‐up)					0.001
Yes	341 (69.0)	149 (78.4)	46 (69.7)	146 (61.3)	
No	153 (31.0)	41 (21.6)	20 (30.3)	92 (38.7)	
Complete self‐reported PrEP adherence (week 4)^g^					<0.001
Yes	154 (31.2)	79 (41.6)	24 (36.4)	51 (21.4)	
No	340 (68.8)	111 (58.4)	42 (63.6)	187 (78.6)	
Any PrEP‐related gastrointestinal symptoms (week 4)^h^					0.036
Yes	170 (43.1)	80 (50.3)	16 (32.0)	74 (40.0)	
No	224 (56.9)	79 (49.7)	34 (68.0)	111 (60.0)	

^a^ Last 12 months.

^b^ For Brazil and Mexico: last 6 months, for Peru: last 3 months.

^c^ Last 6 months.

^d^ Last 3 months.

^e^ Stimulant use was defined as use of any: club drugs (e.g. ecstasy, LSD and GHB), cocaine (powder, crack or paste).

^f^ Attending the 4‐week visit and two or more visits in 52 weeks of follow‐up.

^g^ Report of missing any pill in the previous 30 days.

^h^ Measured among the *n* = 395 (80.0%) of individuals who returned for a 4‐week visit.

^i^ Fisher's exact test for count data with simulated *p*‐value.

Source: ImPrEP Study (2018–2021).

Abbreviations: CAS, condomless anal sex; IQR, interquartile range; PrEP, pre‐exposure prophylaxis; TGW, transgender women.

Overall, TGW were followed‐up for 274.5 person‐years and five HIV seroconversions occurred resulting in an overall HIV incidence rate of 1.82 (95% CI: 0.76–4.38) per 100 person‐years. The HIV incidence rate was 3.80 (95% CI: 1.58–9.13) in Peru, while no HIV cases were observed in Brazil or Mexico. Incidence rate among TGW aged 18–24 and 25–34 years was twice as high compared to TGW aged >34 years (Table 2).

**Table 2:** PrEP use and HIV incidence overall and stratified per country and age

	HIV infection, *n*	Person‐years of follow‐up	Incidence rate per 100 person‐years (95% CI)
Overall	5	274.5	1.82 (0.59–4.25)
Country			
Brazil	0	116.4	0.00 (0.00–3.17)
Mexico	0	26.5	0.00 (0.00–13.92)
Peru	5	131.6	3.80 (1.23–8.87)
Age (years)			
18–24	2	72.3	2.77 (0.34–9.99)
25–34	3	110.3	2.72 (0.56–7.95)
>34	0	91.9	0.00 (0.00–4.01)

Abbreviations: CI, confidence interval; PrEP, pre‐exposure prophylaxis.

**Figure 2:**
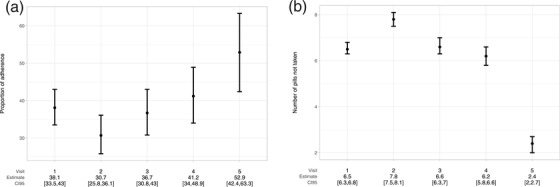
(a) Proportion of complete self‐reported adherence. (b) Number of PrEP pills not taken by visit.

Overall, 101 (20.5%) TGW attended one follow‐up visit, 66 (13.4%) two, 72 (14.6%) three, 80 (16.2%) four, while only 85 (17.2%) completed all five visits. A total of 237 (48.6%) had long‐term PrEP engagement, higher in Brazil (58.8%) than Peru (44.7%) and Mexico (33.3%). Complete self‐reported PrEP adherence increased over time among TGW who attended follow‐up visits (38.1% [95% CI: 33.5–43.0] at visit 1 (4 weeks) vs. 52.9% [95% CI: 42.4–63.3] at visit 5 (∼52 weeks); Figure 2a). Similarly, the number of pills not taken decreased during follow‐up (6.5 [95% CI: 6.3–6.8] pills at visit 1 vs. 2.4 [95% CI: 2.0–2.7] at visit 5; Figure 2b). PrEP‐related gastrointestinal symptoms at week 4 were reported by 43.1%, with higher proportion among Brazilian TGW (50.3%).

In the final multivariate model, long‐term PrEP engagement was higher among TGW who had complete self‐reported PrEP adherence at week 4 (aOR: 2.94 [95% CI: 1.88–4.63]) (Table 3). Long‐term engagement was lower among TGW reporting CAS with partner(s) of unknown HIV status (aOR: 0.52 [95% CI: 0.34–0.81]), who had migrated (aOR: 0.54 [95% CI: 0.34–0.84]) and were from Mexico (aOR: 0.28 [95% CI: 0.14–0.53]). After adjustment, more than secondary education and seeking PrEP as the main reason to attend the service were no long significant. However, the direction of the association remained the same and both had borderline p‐values and confidence intervals.

**Table 3:** Factors associated with long‐term PrEP engagement

	Long‐term PrEP engagement^a^		Bivariate analyses		Multivariate analysis	
	Yes *N* = 237 (%)	No *N* = 251 (%)	OR (95% CI)	*p*‐value	aOR (95% CI)	*p*‐value
Country						
Brazil	110 (58.8)	77 (41.2)	Ref.	Ref.		
Mexico	22 (33.3)	44 (66.7)	**0.35 (0.19, 0.62)**	**<0.001**	**0.28 (0.14, 0.53)**	**<0.001**
Peru	105 (44.5)	130 (55.5)	**0.57 (0.38, 0.83)**	**0.004**	0.91 (0.54, 1.56)	0.740
Age (years)						
18–24	58 (43.3)	76 (56.7)	0.81 (0.52, 1.27)	0.360	0.89 (0.55, 1.46)	0.650
25–34	97 (47.1)	109 (52.9)	Ref.	Ref.	Ref.	
>34	82 (55.8)	66 (44.6)	1.49 (0.97, 2.31)	0.070	1.22 (0.75, 1.97)	0.430
Race or skin colour						
Other race	179 (46.6)	205 (53.4)	0.87 (0.55, 1.39)	0.560	NA	NA
White	58 (55.8)	46 (44.2)	Ref.	Ref.	NA	NA
Education						
Primary (complete or incomplete)	17 (37.0)	29 (63.0)	0.73 (0.37, 1.39)	0.340	0.93 (0.44, 1.92)	0.850
Secondary (complete or incomplete)	128 (44.6)	159 (55.4)	Ref.	Ref.	Ref.	Ref.
More than secondary	92 (59.4)	63 (40.6)	**2.05 (1.35, 3.14)**	**<0.001**	1.55 (0.98, 2.46)	0.063
Migration						
Yes	53 (38.7)	84 (61.3)	**0.55 (0.36, 0.83)**	**0.005**	**0.54 (0.34, 0.84)**	**0.007**
No	171 (51.8)	159 (48.2)	Ref.	Ref.	Ref.	Ref.
Main reason to attend the service						
Searching for PrEP	171 (53.4)	149 (46.6)	**1.82 (1.13, 2.95)**	**0.014**	1.59 (0.95, 2.67)	0.081
Other	66 (39.3)	102 (60.7)	Ref.	Ref.	Ref.	Ref.
Number of cisgender man or/and TGW sex partners^b^						
<5	54 (52.9)	48 (47.1)	Ref.	Ref.	NA	NA
5–10	46 (55.4)	37 (44.6)	1.24 (0.68, 2.26)	0.480	NA	NA
>10	137 (45.2)	166 (54.8)	0.73 (0.46, 1.16)	0.190	NA	NA
CAS^c^						
Yes	217 (49.3)	223 (50.7)	1.34 (0.72, 2.53)	0.360	NA	NA
No	20 (41.7)	28 (58.3)	Ref.	Ref.	NA	NA
Receptive CAS^c^						
Yes	208 (49.4)	213 (50.6)	1.26 (0.74, 2.16)	0.400	NA	NA
No	29 (43.3)	38 (56.7)	Ref.	Ref.	NA	NA
CAS with partner(s) known to be living with HIV^c^						
Yes	13 (65.0)	7 (35.0)	1.22 (0.46, 3.47)	0.700	0.98 (0.33, 3.08)	0.970
No	87 (58.0)	63 (42.0)	Ref.	Ref.	Ref.	Ref.
I don't know	137 (43.1)	181 (56.9)	**0.52 (0.35, 0.78)**	**0.002**	**0.52 (0.34, 0.81)**	**0.004**
Transactional sex^c^						
Yes	158 (45.3)	191 (54.7)	**0.65 (0.43, 0.97)**	**0.038**	0.96 (0.60, 1.52)	0.85
No	79 (56.8)	60 (43.2)	Ref.	Ref.	Ref.	Ref.
Binge drinking^d^						
Yes	158 (47.7)	173 (52.3)	0.92 (0.62, 1.36)	0.660	NA	NA
No	79 (50.3)	78 (49.7)	Ref.	Ref.	NA	NA
Stimulant use^d^, ^e^						
Yes	49 (49.0)	51 (51.0)	0.98 (0.62, 1.55)	0.940	NA	NA
No	188 (48.5)	200 (51.5)	Ref.	Ref.	NA	NA
Complete self‐reported PrEP adherence at week 4^f^						
Yes	104 (67.5)	50 (32.5)	**3.09 (2.05, 4.72)**	**<0.001**	**2.94 (1.88, 4.63)**	**<0.001**
No	133 (39.8)	201 (60.2)	Ref.	Ref.	Ref.	Ref.

Bold indicates *p* < 0.05.

Abbreviations: CAS, condomless anal sex; CI, confidence interval; OR, odds ratio; PrEP, pre‐exposure prophylaxis; TGW, transgender women.

^a^ Attending the 4‐week visit and two or more visits in 52 weeks of follow‐up. Six participants were excluded from this analysis due to HIV seroconversion (*n* = 2), medical concerns (*n* = 1) and adverse effects (*n* = 3), all occurring before the third follow‐up visit.

^b^ For Brazil and Mexico: last 3 months, for Peru: last 6 months.

^c^ Last 6 months.

^d^ Last 3 months.

^e^ Stimulant use was defined as use of any: club drugs (e.g. ecstasy, LSD and GHB), cocaine (powder, crack or paste).

^f^ Report of any missing pill in the previous 30 days.

In the final multivariate model, complete self‐reported PrEP adherence was lower among Mexican (aOR: 0.48 [95% CI: 0.28–0.82]) and Peruvian TGW (aOR: 0.29 [95% CI: 0.21–0.41]) compared to those from Brazil. TGW reporting PrEP‐related symptoms also had lower self‐reported adherence (aOR: 0.63 [95% CI: 0.42–0.92]). TGW aged >34 years (aOR: 1.61 [95% CI: 1.10–2.34]) compared to those aged 25–34 years; TGW reporting CAS with a partner of unknown HIV status also had higher self‐reported adherence (aOR: 1.47 [95% CI: 1.01–2.12]; and those who completed more than secondary education (aOR: 1.55 [95% CI: 1.10–2.19]) compared to secondary education had higher odds of complete self‐reported PrEP adherence (Table 4).

**Table 4:** Factors associated with complete self‐reported adherence during the study

	Bivariate analyses		Multivariate analysis	
	OR (95% CI)	*p*‐value	aOR (95% CI)	*p*‐value
Country^a^				
Brazil	Ref.	Ref.	Ref.	Ref.
Mexico	**0.52 (0.31, 0.86)**	**0.011**	**0.48 (0.28, 0.82)**	**0.007**
Peru	**0.32 (0.23, 0.44)**	**<0.001**	**0.29 (0.21, 0.41)**	**<0.001**
Age (years)^a^				
18–24	0.73 (0.49, 1.10)	0.12	0.76 (0.51, 1.14)	0.160
25–34	Ref.	Ref.	Ref.	Ref.
>34	**1.69 (1.16, 2.45)**	**0.006**	**1.61 (1.10, 2.34)**	**0.014**
Race or skin color^a^				
Other race	0.97 (0.66, 1.44)	0.88	NA	NA
White	Ref.	Ref.	NA	NA
Education^a^				
Primary (complete or incomplete)	0.82 (0.43, 1.57)	0.55	0.85 (0.45, 1.61)	0.620
Secondary (complete or incomplete)	Ref.	Ref.	Ref.	Ref.
More than secondary	**1.60 (1.15, 2.23)**	**0.005**	**1.55 (1.10, 2.19)**	**0.013**
Migration^a^				
Yes	1.08 (0.75, 1.55)	0.67	NA	NA
No	Ref.	Ref.	NA	NA
Main reason to attend the service^a^				
Seeking PrEP	1.11 (0.73, 1.69)	0.62	NA	NA
Other	Ref.	Ref.	NA	NA
Number of cisgender man or/and TGW sex partners^b^				
<5	Ref.	Ref.	NA	NA
5–10	1.35 (0.87, 2.09)	0.18	NA	NA
>10	1.29 (0.89, 1.88)	0.19	NA	NA
Condomless anal sex^c^				
Yes	0.89 (0.61, 1.31)	0.55	NA	NA
No	Ref.	Ref.	NA	NA
Condomless receptive anal sex^c^				
Yes	0.96 (0.68, 1.37)	0.83	NA	NA
No	Ref.	Ref.	NA	NA
Condomless sex with partner(s) known to be living with HIV^c^				
Yes	1.07 (0.48, 2.39)	0.87	1.03 (0.45, 2.38)	0.940
No	Ref.	Ref.	Ref.	Ref.
I don't know	1.39 (0.97, 1.98)	0.072	**1.47 (1.01, 2.12)**	**0.047**
Transactional sex^a^				
Yes	0.80 (0.57, 1.13)	0.21	NA	NA
No	Ref.	Ref.	NA	NA
Binge drinking^c^				
Yes	0.84 (0.60, 1.31)	0.30	NA	NA
No	Ref.	Ref.	NA	NA
Stimulant use^c^, ^d^				
Yes	0.90 (0.61, 1.31)	0.57	NA	NA
No	Ref.	Ref.	NA	NA
Any symptom related to PrEP^e^				
Yes	**0.61 (0.41, 0.90)**	**0.012**	**0.63 (0.42, 0.92)**	**0.019**
No	Ref.	Ref.	Ref.	Ref.

Bold indicates *p* < 0.05.

Abbreviations: aOR, adjusted OR; 95% CI, 95% confidence interval; NA, not applicable; OR, odds ratio.

^a^ Data collected at enrolment.

^b^ Collected every visit (except at week 4) and referred to the previous 3 months. For week 4, we considered information collected at enrolment (for Brazil and Mexico: previous 3 months, for Peru: previous 6 months).

^c^ Collected every visit (except at week 4) and referred to the previous 3 months. For week 4, we considered information collected at enrolment (previous 6 months for all variables, except number of sex partners in Brazil and Mexico and substance use [previous 3 months]).

^d^ Stimulant use was defined as use of any: club drugs (e.g. ecstasy, LSD and GHB), cocaine (powder, crack or paste).

^e^ Any of the following: diarrhoea, flatulence, nausea, vomit, abdominal pain or other. Collected every visit since week 4 and referred to any symptom related to PrEP since the previous visit.

## 4 DISCUSSION

TGW enrolled in the ImPrEP study were able to safely initiate same‐day oral PrEP. The ImPrEP study is the first to evaluate PrEP implementation among Latin‐American TGW and includes a large cohort of TGW, the largest in LMICs with results reported separately from MSM to our knowledge. Long‐term PrEP engagement and self‐reported adherence were low and associated with underlying socio‐demographic characteristics, such as age and education. Our data corroborate the finding that early adherence as measured by self‐report at week 4 is associated with higher likelihood of long‐term PrEP engagement [31]. Although HIV prevalence among TGW is high in Latin America, no HIV incident cases were observed in Brazil and Mexico in a context with PrEP availability at no cost to the user. Conversely, HIV incidence in Peru was high, especially among younger TGW.

In our analysis, less than half of TGW (47.6%) remained engaged in PrEP over the year of follow‐up, lower than observed for MSM included in the ImPrEP study (*p*<0.001) [52] and reflecting long‐term PrEP engagement among TGW in past studies [31, 36]. Long‐term PrEP engagement was lower in Mexico, while complete self‐reported PrEP adherence was lower in Peru, indicating gaps in PrEP services in these settings. Peru and Mexico have adopted trans‐specific guidelines for care [37, 53], but the promises of services tailored to the needs of TGW remain a goal rather than a reality. More than half of TGW (49/89, 55%) enrolled in a Peruvian study to provide support for PrEP users were lost to follow‐up in a short period (3 months) [36]. In Brazil, high retention (111/130, 85%) was observed in the PrEParadas study, a PrEP demonstration study designed for TGW, including gender‐affirming care environment implemented at the study site and TGW peer‐educators [39]; nonetheless, PrEP adherence decreased over time, especially among TGW with lower education [39]. TGW consistently have more difficulties in engaging in prevention and treatment services, reflecting their underlying vulnerabilities and the poor adaptation of services to their needs. Novel HIV prevention strategies will only succeed if health services are acceptable and accessible to TGW [3].

Although long‐term PrEP engagement and self‐reported PrEP adherence are related outcomes, the variables associated with each were distinct. Self‐reported PrEP adherence was higher among TGW with post‐secondary education. Lower education was previously associated with low PrEP adherence among Brazilian TGW [39]. Education level is also an important aspect related to HIV outcomes among people living with HIV [54, 55]. Notably, long‐term PrEP engagement was lower among TGW who had migrated. Internal and external migration seeking better opportunities is common in LMICs. TGW usually migrate to larger cities probably aiming for less stigma and more life opportunities [56]. In a Brazilian study that enrolled 345 TGW, 40% were internal migrants [30]. Although we have not measured income in this study, these results suggest that additional social and financial support might increase PrEP adherence and engagement among TGW with high socio‐economic vulnerability.

Interest in PrEP, based on complete adherence at the week 4 visit was associated with long‐term PrEP engagement. Additionally, PrEP as the main reason for attending the service was borderline significant. In South Africa, PrEP education emerged as an urgent matter for TGW [57]. Expanding PrEP literacy among TGW communities, including knowledge about PrEP benefits, duration of side effects and importance of adherence, is essential for achieving better PrEP outcomes. Targeted adherence‐supporting interventions and peer support activities may be especially important [36] for TGW who are offered PrEP but were not looking for PrEP, those who are younger and with lower education levels, helping to improve PrEP engagement and adherence. TGW remain highly vulnerable to HIV and public health programmes offering PrEP should include tailored support for this population to bolster adherence and engagement to services.

The country‐level differences observed for long‐term PrEP engagement and self‐reported adherence likely reflect underlying distinct public health systems and TGW populations included in each setting. Lower long‐term PrEP engagement in Mexico may reflect the fact that most study sites had stronger connections with MSM, which might made TGW feel less included and hence less informed. In Peru, TGW reported consistently lower adherence compared to the other countries. Compared to Brazil and Mexico, TGW from Peru had lower educational levels and were the least likely to have enrolled seeking PrEP, suggesting lower PrEP awareness, and ultimately impacting their PrEP adherence. Differences in the characteristics of the enrolled TGW may have contributed to their lower adherence and consequently higher HIV incidence, even though not all evaluated variables were significant on their own.

Our findings on long‐term PrEP engagement reflect the difficulties that TGW face to remain engaged in services. Efforts should be taken to retain TGW, including support to their existing social networks [36, 58, 59] and building on the experience of TGW who do return for follow‐up visits. TGW remain highly marginalized, as evidenced by the rates of transphobia in Latin America. Out of the 375 murders of trans people reported between October 2020 and September 2021 worldwide, the great majority (83%) occurred in Latin America; and Brazil and Mexico are in the top of the list [60]. Intersecting social vulnerabilities must be acknowledged when planning PrEP services for TGW.

Our study has limitations. First, ImPrEP was not designed to specifically assess outcomes among TGW, and, therefore, measures of key importance for this population were not evaluated. Data on FHT use are not available for most of TGW participants, so we could not include this information in this analysis. Additional qualitative studies to assure understanding of the factors influencing PrEP adherence and engagement among TGW may be needed. Our results are not informative of PrEP uptake, given the study design, study screening only occurred among TGW who expressed an interest in participating. The study inclusion criteria focused on enrolling individuals who could benefit from PrEP, but not all potentially eligible individuals wanted to be screened. Data on PrEP refusal were not collected. Inclusion of fewer TGW from Mexico and data from various sites within a relatively small sample may limit cross country comparisons.

Although self‐reported adherence can be limited by different biases, such as recall, response or social desirability bias, which may overestimate adherence [61], neutral assessment (assessment conducted by non‐clinical/non‐counselling staff trained to collect adherence information without judgement or negative consequences) [62] can minimize these biases and is recommended to ensure the quality of self‐report [49]. Previous analyses from a Brazilian PrEP study have shown that, self‐reported adherence can discriminate participants with and without protective TDF‐FTC levels [49, 50]. In a recent study from New York (USA), self‐reported PrEP adherence has shown to be accurate and a valid indicator of PrEP uptake [63]. Our analysis of self‐reported PrEP adherence used a very stringent definition, requiring only individuals who reported taking all pills in 30 days to be categorized as adherent. However, the number of missing pills reported by visit (Figure 2a) led to sufficient number of pills required for protection (i.e. 4 pills per week, which would provide sufficient protection) [20]. Additionally, the average number of missing pills (Figure 2b) ranged from 2 to 8 within the past 30 days and decreased over time. Importantly, self‐reported adherence is based on TGW who attended the service and, therefore, does not include those who missed visits.

## 5 CONCLUSIONS

Although TGW ere willing to be enrolled in ImPrEP and remained on oral PrEP during short‐term follow‐up, long‐term PrEP engagement and PrEP adherence were limited. HIV incidence remained high in Peru despite the availability of PrEP free of charge throughout the study. A successful HIV prevention agenda among TGW considering country or region particularities will need to address social and financial barriers and include trans‐tailored interventions supporting PrEP education, engagement and adherence. Long‐acting PrEP may be particularly useful for this population.

## COMPETING INTERESTS

KAK reports employment at Universidad Peruana Cayetano Heredia and University of California, Los Angeles. All other authors report no potential competing interests.

## AUTHORS’ CONTRIBUTIONS

VGV, CFC, BG and HV‐R conceived and designed the ImPrEP study. BG conceived and supervised the current analysis and manuscript preparation. KAK and TST interpreted the findings and drafted the manuscript. RIM, ICL and MC did the statistical analyses. EMJ, BH, JVG, MB, CP, SB‐A and HV helped with data acquisition, interpretation of the findings and drafting the manuscript. GM and AR were involved in revising the manuscript for important intellectual content. All authors read and approved the final manuscript.

### ImPrEP Study Group

#### Brazil

Marcus V. de Lacerda, Alessandro Farias, J. David Urbaez‐Brito, Polyana d'Albuquerque, Claudio Palombo, Lilian Lauria, Josué Lima, Paulo Ricardo de Alencastro, Ronaldo Zonta, Raquel Keiko de Luca Ito, José Valdez Madruga, João L. de Benedetti, Fabio V. Maria, Paula M. Luz, Lucilene Freitas, Kim Geraldo, Monica Derrico, Sandro Nazer, Tania Kristic, Renato Girade (*in memoriam*), Renato Lima, Antônio R. de Carvalho, Julio Moreira, Carla Rocha, Pedro Leite, Marcio Lessa, Marilia Santini, Daniel R. B. Bezerra, Cleo de Oliveira Souza, Jacinto Corrêa, Marcelo Alves, Carolina Souza, Camilla Portugal, Mônica dos Santos Valões, Gabriel Lima Mota, Joyce Alves Gomes, Cynthia Ferreira Lima Falcão, Fernanda Falcão Riberson, Luciano Melo, Talita Andrade Oliva, Agnaldo Moreira de Oliveira Júnior, Bruna Fonseca, Leonor Henriette de Lannoy, Ludymilla Anderson Santiago Carlos, João Paulo da Cunha, Sonia Maria de Alencastro Coracini, Thiago Oliveira Rodrigues, Emília Regina Scharf Mettrau, Kelly Vieira Meira; Heder Tavares, Ana Paula Nunes Viveiros Valeiras, Taiane Miyake Alves de Carvalho Rocha, Alex Amorim, Patrícia Sabadini, Luiz Gustavo Córdoba, Caio Gusmão, Erika Faustino, Julia Soares da Silva Hansen, Agatha Mirian Cunha, Neuza Uchiyama Nishimura, Jaime Eduardo Flygare Razo Prereira dos Santos, Aline Barnabé Cano, Willyam Magnum Telles Dias, Magô Tonhon, Tania Regina Rezende, Alex Gomes, Eloá dos Santos Rodrigues, Maria das Dores Aires Carneiro, Alexandre Castilho, Mariana Carvalho.

#### Mexico

Steven Díaz, Centli Guillén, Lorena Hernández, Rebeca Robles, María Elena Medina‐Mora, Marcela González, Ivonne Huerta Icelo, Araczy Martinez Davalos, Diego Cerecero Garcia, José Gomez Castro, Santiago Aguilera Mijares, Luis Obed Ocampo Valdez, Alma Cruz, Arantxa Colchero Aragones.

#### Peru

Gino Calvo, Silver Vargas, Oliver Elorreaga, Ximena Gutierrez, Fernando Olivos, Damaris Caviedes, Daniela Oquendo, Eduardo Juárez, Jazmin Qquellon, Francesca Vasquez, Jean Pierre Jiron, Sonia Flores, Karen Campos

#### ImPrEP Programme Officers

Heather L. Ingold, Anna Hellstrom

### ImPrEP Study Sites

#### Brazil

Fundação de Medicina Tropical (Manaus, Amazonas), Hospital Universitário Oswaldo Cruz (Recife, Pernambuco), CEDAP – Centro Estadual Especializado em Diagnóstico, Assistência e Pesquisa (Salvador, Bahia), Hospital Dia Asa Sul (Brasília, Distrito Federal), Instituto Nacional de Infectologia Evandro Chagas, Fundação Oswaldo Cruz INI‐Fiocruz (Rio de Janeiro), Hospital Municipal Rocha Maia (Rio de Janeiro), Hospital Municipal Carlos Tortelly (Niterói, Rio de Janeiro), Centro de Referência em DST/AIDS‐ AMDA (Campinas, São Paulo), Centro de Referência e Treinamento em DST/AIDS – CRT‐SP (São Paulo), SAE DST/AIDS – CECI (São Paulo), SAE DST/AIDS – Fidélis Ribeiro (São Paulo), SAE Adulto (Santos, São Paulo), Poli Centro (Florianópolis, Santa Catarina), SAT – Sanatório Partenon (Porto Alegre, Rio Grande do Sul).

#### Mexico

Clínica Especializada Condesa (Cuauhtémoc, Mexico City), Fundación Unidos por un México Vivo A.C. (Cuauhtémoc, Mexico City), Comité Humanitario de Esfuerzo Compartido Contra El Sida A.C. (Guadalajara, Jalisco), Solidaridad Ed Thomas A.C. (Puerto Vallarta, Jalisco).

#### Peru

Centro de Referencia de Infecciones de Transmisión Sexual del Centro Materno Infantil San José (Lima), Centro de Referencia de Infecciones de Transmisión Sexual del Centro Materno Infantil Tahuantinsuyo Bajo (Lima), Centro de Referencia de Infecciones de Transmisión Sexual del Centro de Salud Alberto Barton (Callao), Centro de Referencia de Infecciones de Transmisión Sexual de Caja de Agua (Lima), Centro de Referencia de Infecciones de Transmisión Sexual del Hospital Amazónico Pucallpa (Ucayali), Centro de Referencia de Infecciones de Transmisión Sexual del Hospital La Caleta Chimbote (Ancash), Centro de Referencia de Infecciones de Transmisión Sexual del Hospital Regional Ica (Ica), Centro de Referencia de Infecciones de Transmisión Sexual del Hospital Regional Trujillo (La Libertad), Investigaciones Médicas en Salud, INMENSA (Lima), Centro de Referencia de Infecciones de Transmisión Sexual del Hospital San Juan De Dios, Pisco (Ica).

## FUNDING

BG and TST are funded by the National Council of Technological and Scientific Development (CNPq) and Carlos Chagas Filho Foundation for Research Support in the State of Rio de Janeiro (FAPERJ).

## Data Availability

Data will be available upon reasonable request and will be approved by ImPrEP coordination team.

## References

[1] Reisner SL , Poteat T , Keatley J , Cabral M , Mothopeng T , Dunham E , et al. Global health burden and needs of transgender populations: a review. Lancet. 2016;388(10042):412–36. 27323919 10.1016/S0140-6736(16)00684-X PMC7035595

[2] Baral SD , Poteat T , Stromdahl S , Wirtz AL , Guadamuz TE , Beyrer C . Worldwide burden of HIV in transgender women: a systematic review and meta‐analysis. Lancet Infect Dis. 2013;13(3):214–22. 23260128 10.1016/S1473-3099(12)70315-8

[3] Poteat T , Scheim A , Xavier J , Reisner S , Baral S . Global epidemiology of HIV infection and related syndemics affecting transgender people. J Acquir Immune Defic Syndr. 2016;72(Suppl 3):S210–9. 27429185 10.1097/QAI.0000000000001087 PMC4969059

[4] Stutterheim SE , van Dijk M , Wang H , Jonas KJ . The worldwide burden of HIV in transgender individuals: an updated systematic review and meta‐analysis. PLoS One. 2021;16(12):e0260063. 34851961 10.1371/journal.pone.0260063 PMC8635361

[5] Bastos FI , Bastos LS , Coutinho C , Toledo L , Mota JC , Velasco‐de‐Castro CA , et al. HIV, HCV, HBV, and syphilis among transgender women from Brazil: assessing different methods to adjust infection rates of a hard‐to‐reach, sparse population. Medicine (Baltimore). 2018;97(1S Suppl 1):S16–24. 29794601 10.1097/MD.0000000000009447 PMC5991532

[6] Colchero MA , Cortes‐Ortiz MA , Romero‐Martinez M , Vega H , Gonzalez A , Roman R , et al. HIV prevalence, sociodemographic characteristics, and sexual behaviors among transwomen in Mexico City. Salud Pública Méx. 2015;57(Suppl 2):s99–106. 26545137 10.21149/spm.v57s2.7596

[7] Silva‐Santisteban A , Raymond HF , Salazar X , Villayzan J , Leon S , McFarland W , et al. Understanding the HIV/AIDS epidemic in transgender women of Lima, Peru: results from a sero‐epidemiologic study using respondent driven sampling. AIDS Behav. 2012;16(4):872–81. 21983694 10.1007/s10461-011-0053-5

[8] Badgett MVL , Nezhad S , Waaldijk K , Rodgers YM . The relationship between LGBT inclusion and economic development: an analysis of emerging economies. 2014. https://www.usaid.gov/sites/default/files/documents/15396/lgbt‐inclusion‐and‐development‐november‐2014.pdf. Accessed 15 July 2022.

[9] Silva MAD , Luppi CG , Veras M . Work and health issues of the transgender population: factors associated with entering the labor market in the state of Sao Paulo, Brazil. Cien Saude Colet. 2020;25(5):1723–34. 32402024 10.1590/1413-81232020255.33082019

[10] Silva‐Santisteban A , Raymond HF , Salazar X , Villayzan J , Leon S , McFarland W , et al. Understanding the HIV/AIDS epidemic in transgender women of Lima, Peru: results from a sero‐epidemiologic study using respondent driven sampling. AIDS Behav. 2012;16(4):872–81. 21983694 10.1007/s10461-011-0053-5

[11] Cheney MK , Gowin MJ , Taylor EL , Frey M , Dunnington J , Alshuwaiyer G , et al. Living outside the gender box in Mexico: testimony of transgender Mexican asylum seekers. Am J Public Health. 2017;107(10):1646–52. 28817317 10.2105/AJPH.2017.303961 PMC5607674

[12] Mendes WG , Silva C . Homicide of lesbians, gays, bisexuals, travestis, transexuals, and transgender people (LGBT) in Brazil: a spatial analysis. Cien Saude Colet. 2020;25(5):1709–22. 32402041 10.1590/1413-81232020255.33672019

[13] Suarez EB , Logie C , Arocha JF , Sanchez H , Shokirova T . Contesting everyday violence: resilience pathways of gay and transgender youth in Peru. Glob Public Health. 2021;16(5):706–28. 33284733 10.1080/17441692.2020.1856397

[14] Murphy EC , Segura ER , Lake JE , Huerta L , Perez‐Brumer AG , Mayer KH , et al. Intimate partner violence against transgender women: prevalence and correlates in Lima, Peru (2016–2018). AIDS Behav. 2020;24(6):1743–51. 31720954 10.1007/s10461-019-02728-w PMC7214207

[15] Leite BO , de Medeiros DS , Magno L , Bastos FI , Coutinho C , de Brito AM , et al. Association between gender‐based discrimination and medical visits and HIV testing in a large sample of transgender women in northeast Brazil. Int J Equity Health. 2021;20(1):199. 34488781 10.1186/s12939-021-01541-z PMC8422640

[16] Sevelius J , Murray LR , Martinez Fernandes N , Veras MA , Grinsztejn B , Lippman SA . Optimising HIV programming for transgender women in Brazil. Cult Health Sex. 2019;21(5):543–58. 30378463 10.1080/13691058.2018.1496277 PMC6483864

[17] Passaro RC , Segura ER , Lama JR , Sanchez J , Lake JE , Shoptaw S , et al. High‐risk, but hidden: binge drinking among men who have sex with men and transgender women in Lima, Peru, 2012–2014. Subst Use Misuse. 2020;55(3):399–404. 31682179 10.1080/10826084.2019.1681451 PMC7002235

[18] Wylie K , Knudson G , Khan SI , Bonierbale M , Watanyusakul S , Baral S . Serving transgender people: clinical care considerations and service delivery models in transgender health. Lancet. 2016;388(10042):401‐11. 27323926 10.1016/S0140-6736(16)00682-6

[19] Grant RM , Lama JR , Anderson PL , McMahan V , Liu AY , Vargas L , et al. Preexposure chemoprophylaxis for HIV prevention in men who have sex with men. N Engl J Med. 2010;363(27):2587–99. 21091279 10.1056/NEJMoa1011205 PMC3079639

[20] Anderson PL , Glidden DV , Liu A , Buchbinder S , Lama JR , Guanira JV , et al. Emtricitabine‐tenofovir concentrations and pre‐exposure prophylaxis efficacy in men who have sex with men. Sci Transl Med. 2012;4(151):151ra25. 10.1126/scitranslmed.3004006 PMC3721979 22972843

[21] Coelho LE , Torres TS , Veloso VG , Landovitz RJ , Grinsztejn B . Pre‐exposure prophylaxis 2.0: new drugs and technologies in the pipeline. Lancet HIV. 2019;6(11):e788–99. 31558423 10.1016/S2352-3018(19)30238-3

[22] Deutsch MB , Glidden DV , Sevelius J , Keatley J , McMahan V , Guanira J , et al. HIV pre‐exposure prophylaxis in transgender women: a subgroup analysis of the iPrEx trial. Lancet HIV. 2015;2(12):e512–9. 26614965 10.1016/S2352-3018(15)00206-4 PMC5111857

[23] Cottrell ML , Prince HMA , Schauer AP , Sykes C , Maffuid K , Poliseno A , et al. Decreased tenofovir diphosphate concentrations in a transgender female cohort: implications for human immunodeficiency virus preexposure prophylaxis. Clin Infect Dis. 2019;69(12):2201–4. 30963179 10.1093/cid/ciz290 PMC7188232

[24] Hiransuthikul A , Janamnuaysook R , Himmad K , Kerr SJ , Thammajaruk N , Pankam T , et al. Drug–drug interactions between feminizing hormone therapy and pre‐exposure prophylaxis among transgender women: the iFACT study. J Int AIDS Soc. 2019;22(7):e25338. 31298497 10.1002/jia2.25338 PMC6625338

[25] Shieh E , Marzinke MA , Fuchs EJ , Hamlin A , Bakshi R , Aung W , et al. Transgender women on oral HIV pre‐exposure prophylaxis have significantly lower tenofovir and emtricitabine concentrations when also taking oestrogen when compared to cisgender men. J Int AIDS Soc. 2019;22(11):e25405. 31692269 10.1002/jia2.25405 PMC6832671

[26] Cattani VB , Jalil EM , Eksterman LF , Torres T , Cardoso SW , Castro CRV , et al. Impact of feminizing hormone therapy on tenofovir and emtricitabine plasma pharmacokinetics: a nested drug–drug interaction study in a cohort of Brazilian transgender women using HIV pre‐exposure prophylaxis. J Antimicrob Chemother. 2022;dkac229. 10.1093/jac/dkac229 PMC9525093 35815666

[27] Gomez GB , Borquez A , Caceres CF , Segura ER , Grant RM , Garnett GP , et al. The potential impact of pre‐exposure prophylaxis for HIV prevention among men who have sex with men and transwomen in Lima, Peru: a mathematical modelling study. PLoS Med. 2012;9(10):e1001323. 23055836 10.1371/journal.pmed.1001323 PMC3467261

[28] Ferreira ACG , Coelho LE , Jalil EM , Luz PM , Friedman RK , Guimaraes MRC , et al. Transcendendo: a cohort study of HIV‐infected and uninfected transgender women in Rio de Janeiro, Brazil. Transgend Health. 2019;4(1):107–17. 30972370 10.1089/trgh.2018.0063 PMC6455979

[29] Luz PM , Veloso VG , Grinsztejn B . The HIV epidemic in Latin America: accomplishments and challenges on treatment and prevention. Curr Opin HIV AIDS. 2019;14(5):366–73. 31219888 10.1097/COH.0000000000000564 PMC6688714

[30] Grinsztejn B , Jalil EM , Monteiro L , Velasque L , Moreira RI , Garcia ACF , et al. Unveiling of HIV dynamics among transgender women: a respondent‐driven sampling study in Rio de Janeiro, Brazil. Lancet HIV. 2017;4(4):e169–76. 28188030 10.1016/S2352-3018(17)30015-2 PMC5411266

[31] Grinsztejn B , Hoagland B , Moreira RI , Kallas EG , Madruga JV , Goulart S , et al. Retention, engagement, and adherence to pre‐exposure prophylaxis for men who have sex with men and transgender women in PrEP Brasil: 48 week results of a demonstration study. Lancet HIV. 2018;5(3):e136–45. 29467098 10.1016/S2352-3018(18)30008-0

[32] Del Rio‐Gonzalez AM , Lameiras‐Fernandez M , Modrakovic D , Aguayo‐Romero R , Glickman C , Bowleg L , et al. Global scoping review of HIV prevention research with transgender people: transcending from trans‐subsumed to trans‐centred research. J Int AIDS Soc. 2021;24(9):e25786. 34473421 10.1002/jia2.25786 PMC8412127

[33] Zucchi EM , Couto MT , Castellanos M , Dumont‐Pena E , Ferraz D , Felix Pinheiro T , et al. Acceptability of daily pre‐exposure prophylaxis among adolescent men who have sex with men, travestis and transgender women in Brazil: a qualitative study. PLoS One. 2021;16(5):e0249293. 33945527 10.1371/journal.pone.0249293 PMC8096080

[34] Perez‐Brumer A , Naz‐McLean S , Huerta L , Salazar X , Lama JR , Sanchez J , et al. The wisdom of mistrust: qualitative insights from transgender women who participated in PrEP research in Lima, Peru. J Int AIDS Soc. 2021;24(9):e25769. 34569152 10.1002/jia2.25769 PMC9936804

[35] Jalil EM , Grinsztejn B , Velasque L , Ramos Makkeda A , Luz PM , Moreira RI , et al. Awareness, willingness, and PrEP eligibility among transgender women in Rio de Janeiro, Brazil. J Acquir Immune Defic Syndr. 2018;79(4):445–52. 30142140 10.1097/QAI.0000000000001839 PMC6203608

[36] Clark J , Reisner S , Perez‐Brumer A , Huerta L , Sanchez H , Moriarty K , et al. TransPrEP: results from the pilot study of a social network‐based intervention to support PrEP adherence among transgender women in Lima, Peru. AIDS Behav. 2021;25(6):1873–83. 33385279 10.1007/s10461-020-03117-4 PMC8084919

[37] Salazar X , Nunez‐Curto A , Villayzan J , Castillo R , Benites C , Caballero P , et al. How Peru introduced a plan for comprehensive HIV prevention and care for transwomen. J Int AIDS Soc. 2016;19(3 Suppl 2):20790. 27431469 10.7448/IAS.19.3.20790 PMC4949315

[38] Salazar X , Nunez‐Curto A , Villayzan Aguilar J , Lusquinos M , Motta Ochoa A , Caceres CF . Confluent paths: research and community participation to protect the right to health among transgender women in Peru. Glob Public Health. 2019;14(6–7):954–62. 30929572 10.1080/17441692.2019.1599982

[39] Jalil EM , Torres TS , Luz PM , Monteiro L , Moreira RI , de Castro CRV , et al. Low PrEP adherence despite high retention among transgender women in Brazil: the PrEParadas study. J Int AIDS Soc. 2022;25(3):e25896. 35255199 10.1002/jia2.25896 PMC8901149

[40] PPAH Organization . HIV epidemic and response in Latin America and the Caribbean. 2021. https://www.paho.org/en/documents/hiv‐epidemic‐and‐response‐latin‐america‐and‐caribbean. Accessed 15 July 2022.

[41] Gobierno de Mexico CNplPyCdVyes . Campaña de la Profilaxis pre exposición (PrEP) ¡Para prevenir, éntrale con todo! 2021. https://www.gob.mx/censida/articulos/campana‐de‐la‐profilaxis‐pre‐exposicion‐prep‐para‐prevenir‐entrale‐con‐todo?idiom=es. Accessed 15 July 2022.

[42] Infante C , Sosa‐Rubi SG , Cuadra SM . Sex work in Mexico: vulnerability of male, travesti, transgender and transsexual sex workers. Cult Health Sex. 2009;11(2):125–37. 19140056 10.1080/13691050802431314

[43] Rowan SE , Patel RR , Schneider JA , Smith DK . Same‐day prescribing of daily oral pre‐exposure prophylaxis for HIV prevention. Lancet HIV. 2021;8(2):e114–20. 33128874 10.1016/S2352-3018(20)30256-3

[44] Solomon MM , Lama JR , Glidden DV , Mulligan K , McMahan V , Liu AY , et al. Changes in renal function associated with oral emtricitabine/tenofovir disoproxil fumarate use for HIV pre‐exposure prophylaxis. AIDS. 2014;28(6):851–9. 24499951 10.1097/QAD.0000000000000156 PMC3966916

[45] Torres TS , Hoagland B , Konda K , Vega‐Ramirez EH , Guanira JV , Vermandere H , et al. Impact of COVID‐19 pandemic and pandemic response on cisgender men who have sex with men (MSM) and transwomen in a PrEP cohort from Brazil, Peru and Mexico ‐ ImPrEP study. IAS Conference on HIV Science; 2021.

[46] Hoagland B , Torres TS , Bezerra DRB , Geraldo K , Pimenta C , Veloso VG , et al. Telemedicine as a tool for PrEP delivery during the COVID‐19 pandemic in a large HIV prevention service in Rio de Janeiro‐Brazil. Brazil J Infect Dis. 2020;24(4):360–4. 10.1016/j.bjid.2020.05.004 PMC7261432 32504552

[47] Hoagland B , Torres TS , Bezerra DRB , Benedetti M , Pimenta C , Veloso VG , et al. High acceptability of PrEP teleconsultation and HIV self‐testing among PrEP users during the COVID‐19 pandemic in Brazil. Brazil J Infect Dis. 2021;25(1):101037. 10.1016/j.bjid.2020.11.002 PMC7833416 33285137

[48] (NIAAA) NIoAAaA . Drinking levels defined. 2021. https://www.niaaa.nih.gov/alcohol‐health/overview‐alcohol‐consumption/moderate‐binge‐drinking. Accessed 15 July 2022.

[49] Monteiro Spindola Marins L , Silva Torres T , Luz PM , Moreira RI , Leite IC , Hoagland B , et al. Factors associated with self‐reported adherence to daily oral pre‐exposure prophylaxis among men who have sex with man and transgender women: PrEP Brasil study. Int J STD AIDS. 2021;32(13):1231–41. 34311605 10.1177/09564624211031787

[50] Marins LMS , Torres TS , Leite IDC , Moreira RI , Luz PM , Hoagland B , et al. Performance of HIV pre‐exposure prophylaxis indirect adherence measures among men who have sex with men and transgender women: results from the PrEP Brasil study. PLoS One. 2019;14(8):e0221281. 31430318 10.1371/journal.pone.0221281 PMC6701758

[51] RC Team . R: a language and environment for statistical computing. 2020. www.R‐project.org. Accessed 15 July 2022.

[52] Veloso V , Moreira RI , Konda KA , Hoagland B , Vega‐Ramirez H , Leite IC , et al. PrEP long‐term engagement among MSM and TGW in Latin America ‐ the ImPrEP study. Conference on Retroviruses and Opportunistic Infections; 2022.

[53] Mexico Gd . Protocolo para el acceso sin discriminación a la prestación de servicios de atención médica de las personas lésbico, gay, bisexual, transexual, travesti, transgénero e intersexual y guías de atención específicas. 2020. https://www.gob.mx/cms/uploads/attachment/file/558167/Versi_n_15_DE_JUNIO_2020_Protocolo_Comunidad_LGBTTI_DT_Versi_n_V_20.pdf. Accessed 15 July 2022.

[54] Rodrigues A , Struchiner CJ , Coelho LE , Veloso VG , Grinsztejn B , Luz PM . Late initiation of antiretroviral therapy: inequalities by educational level despite universal access to care and treatment. BMC Public Health. 2021;21(1):389. 33607975 10.1186/s12889-021-10421-8 PMC7893724

[55] Pascom ARP , Meireles MV , Benzaken AS . Sociodemographic determinants of attrition in the HIV continuum of care in Brazil, in 2016. Medicine (Baltimore). 2018;97(1S Suppl 1):S69–74. 29912818 10.1097/MD.0000000000009857 PMC5991540

[56] Gamarel KE , King WM , Mouzoon R , Xie H , Stanislaus V , Iwamoto M , et al. A “tax” on gender affirmation and safety: costs and benefits of intranational migration for transgender young adults in the San Francisco Bay area. Cult Health Sex. 2020;23(12):1763–78. 32924839 10.1080/13691058.2020.1809711 PMC7956137

[57] Poteat T , Malik M , van der Merwe LLA , Cloete A , Adams D , Nonyane BAS , et al. PrEP awareness and engagement among transgender women in South Africa: a cross‐sectional, mixed methods study. Lancet HIV. 2020;7(12):e825–34. 32622370 10.1016/S2352-3018(20)30119-3

[58] Perez‐Brumer AG , Reisner SL , McLean SA , Silva‐Santisteban A , Huerta L , Mayer KH , et al. Leveraging social capital: multilevel stigma, associated HIV vulnerabilities, and social resilience strategies among transgender women in Lima, Peru. J Int AIDS Soc. 2017;20(1):21462. 28362064 10.7448/IAS.20.1.21462 PMC5467605

[59] Bezerra DRB , Jalil CM , Jalil EM , Coelho LE , Carvalheira E , Freitas J , et al. Complementary recruitment strategies to reach men who have sex with men and transgender women: the experience of a large Brazilian HIV prevention service. AIDS Behav. 2022;26(8):2643–52. 35122578 10.1007/s10461-022-03609-5

[60] (TGEU) TE . TVT TMM update trans day of remembrance. 2021. https://transrespect.org/en/tmm‐update‐tdor‐2021/. Accessed 15 July 2022.

[61] Haberer JE . Current concepts for PrEP adherence in the PrEP revolution: from clinical trials to routine practice. Curr Opin HIV AIDS. 2016;11(1):10–7. 26633638 10.1097/COH.0000000000000220 PMC4801217

[62] MacQueen KM , Tolley EE , Owen DH , Amico KR , Morrow KM , Moench T , et al. An interdisciplinary framework for measuring and supporting adherence in HIV prevention trials of ARV‐based vaginal rings. J Int AIDS Soc. 2014;17(3 Suppl 2):19158. 25224617 10.7448/IAS.17.3.19158 PMC4164000

[63] Qasmieh S , Nash D , Gandhi M , Rozen E , Okochi H , Goldstein H , et al. Self‐reported use of HIV pre‐exposure prophylaxis is highly accurate among sexual health clinic patients in New York City. Sex Transm Dis. 2022. 10.1097/OLQ.0000000000001622 PMC9463403 35312670

